# Engineering Sustainable Antimicrobial Release in Silica-Cellulose Membrane with CaCO_3_-Aided Processing for Wound Dressing Application

**DOI:** 10.3390/polym11050808

**Published:** 2019-05-06

**Authors:** Zhi Shen, Ning Cai, Yanan Xue, Vincent Chan, Bo Yu, Jianzhi Wang, Hao Song, Hang Deng, Faquan Yu

**Affiliations:** Key Laboratory for Green Chemical Process of Ministry of Education, Hubei Key Laboratory for Novel Reactor and Green Chemistry Technology, School of Chemical Engineering and Pharmacy, Wuhan Institute of Technology, Wuhan 430073, China; shenzhi20121111@163.com (Z.S.); cnntu@hotmail.com (N.C.); xueyn_wit@163.com (Y.X.); vincent.chan@ku.ac.ae (V.C.); yuboxf@hotmail.com (B.Y.); wangjz15@163.com (J.W.); haosong19920323@163.com (H.S.); denghang7740@gmail.com (H.D.)

**Keywords:** mesoporous silica, antibacterial, drug delivery, wound dressing, cellulose

## Abstract

The sustained release of antimicrobial therapeutics for wound dressing has become an attractive design strategy for prolonging the timespan of wound dressings and for reducing the risk of chronic wound infection. Recently, cellulose-based membrane has become a preferred option of wound dressings for the treatment of burn wounds and skin ulcers. In this work, novel cellulose membrane incorporated with mesoporous silica particles (SBA-15) was developed as an antimicrobial wound dressing with desirable sustained release functionality for targeting persistent bacterial pathogens. Attributed to a coated layer of calcium carbonate (CaCO_3_), SBA-15 particles were free from corrosion in alkaline condition during the preparation of cellulose-based composite membranes. SEM, TEM and BET results showed that the morphology, specific surface area, pore size and pore volume of pristine SBA-15 were preserved after the incorporation of CaCO_3_-coated SBA-15 into the cellulose matrix, while the mesoporous structure of SBA-15 was significantly disrupted without the use of CaCO_3_ coating. The resultant composite membranes containing 30 wt% SBA-15 (denoted as CM-Ca2-SBA(30%)) achieved 3.6 wt% of antimicrobial drug loading. Interestingly, CM-Ca2-SBA(30%) demonstrated the sustained release property of chloramphenicol for 270 h, driven by a two-stage drug release processes of SBA-15/cellulose. The water vapor permeability (WVTR) and swelling properties of composite membranes were shown to have complied with the primary requirements of wound dressing. Antibacterial assays revealed that strong antibacterial activities (144 h) of the composite membranes against *Staphylococcus aureus* and *Eschericia coli* were achieved. All results displayed that the strategy of coating silica with CaCO_3_ helps to obtain cellulose–silica composite membranes with desirable sustained release profiles and strong antibacterial activities. The antibacterial SBA-15/cellulose composite membranes show potential for the application of wound dressing.

## 1. Introduction

As the largest organ of the integumentary system, skin serves as a physiochemical barrier to defend the human body from infections and to restrain the loss of electrolytes and water from other organs [1,2]. Generally, seriously wounded skin must be immediately treated with hydrated wound dressing to prevent further infection and dehydration [3]. Without the protection of wound dressing, the skin’s healing process would be significantly hampered by both bacteria invasion and oxygen/nutrient deficiency [4]. Nowadays, through dermal fibroblast migration and extracellular matrix synthesis, various types of natural and synthetic biomaterials (such as foam, thin film, membrane and hydrogel) have been developed to establish an optimized physiological microenvironment for skin wound healing [5,6].

A clinically effective wound dressing should maintain a moist environment to prevent pathogen invasion, absorb wound exudates and provide ideal biocompatibility [7]. As typical occlusive material dressings used in first aid are impermeable to air and liquid, bacterial infection—which is detrimental to dermal tissue regeneration—can be easily caused by the aggregated exudates from the wound, leading to significant increase of medical expenses [8]. Therefore, porous materials with higher permeability and absorbability is a preferred option for wound dressing application [9]. Macroporous material has demonstrated superior performance in removing excessive exudates efficiently. However, macroporous material prevents neither the invasion of airborne bacteria, nor the dehydration of the wound bed [10]. In contrast to macroporous materials, wound dressing incorporated with micro- and nano-sized pores has been shown to inhibit microorganism invasion effectively and to reduce wound infection [2]. Furthermore, nanofibrous membrane has been shown to provide the desirable topographic characteristics for the adhesion, proliferation and differentiation of fibroblasts inside the wound bed [11]. It was reported that cellulose membranes with interconnected micro- and nano-sized pore structure have demonstrated a superior capacity of water absorption in providing a moist microenvironment that is beneficial to wound healing [12].

Cellulose, which is the main constituent of plant cells, is an odorless, abundant and renewable natural material [13,14]. Moreover, cellulose possesses several attractive physiochemical properties, such as hydrophilicity, biocompatibility, non-immunogenicity, flexibility and biodegradability, as well as a remarkable mechanical property for potential biomedical applications. More importantly, reconstituted cellulose membranes exhibit highly ordered micro- and nano-scale pore structures [15]. Such porous network as mentioned above is commonly regarded as an ideal property for wound dressings, but pristine cellulose does not possess notable antibacterial activity [16,17,18,19]. Recently, loading cellulose with silver (Ag^+^) ions has become an effective way of generating the antibacterial property of wound dressing [18,20,21]. However, exposure of the wound bed to silver ions may induce significant cytotoxicity and delay wound re-epithelialization, limiting their clinical applications [22]. Alternatively, direct encapsulation of antimicrobials within material matrices has emerged as a popular way to enhance the antibacterial activity of the tissue engineering scaffold [23,24]. Sustained release of antimicrobial therapeutics from wound dressing has become an attractive design strategy for prolonging the timespan of wound dressings and for reducing the risk of chronic wound infection. In contrast to its ideal biocompatibility, cellulose membrane has demonstrated low drug loading capacity, as well as nonideal drug release properties (e.g., burst release, short duration, etc.). Although chemical modification has attempted to introduce functional groups on cellulose for improving drug loading and release profiles [25], a number of reports have revealed that chemical contamination would be induced during the functionalization of cellulose [26].

Based on the extensive porous network, high specific surface area and excellent biocompatibility, mesoporous silica has emerged as a common drug carrier [27,28,29,30,31]. When mesoporous silica is incorporated in cellulose membrane, hierarchic pore structure will be attained inside the mesoporous matrix of the cellulose–silica membranes. Recently, a research group prepared a flexible hydrogel membrane from the composite of MCM-41 and carboxymethylcellulose for the controlled release of tetracycline [32]. If drugs are loaded inside the mesoporous matrix of silica subsequent to membrane casting, a two-stage drug diffusion cascade from the silica matrix to the external solution medium will be implemented for improving the drug release mechanism. There, the resulting wound dressing will be expected to reduce the burst release of the drug and achieve a sustained release. More importantly, high drug loading capacity on mesoporous cellulose–silica composite membranes results from the high porosity of mesoporous silica, meeting the clinical requirements. 

NaOH/urea is an efficient and green solvent for sustainable cellulose processing. However, mesoporous silica, including SBA-15, has been known to be vulnerable in a strong alkali environment, which is likely to damage its mesoporous structure during the dispersion of silica particles in cellulose solution during the processing of cellulose-based composite. Therefore, a strategy for protecting SBA-15 against the corrosion of alkali solution is required for the preparation of SBA-15/cellulose composite membranes. CaCO_3_ is a compound that is inert in an alkali environment that can decompose in HCl solution. The objective of this work is to explore the feasibility of coating SBA-15 with a CaCO_3_ layer to protect the mesoporous structure of SBA-15 in the preparation of SBA-15/cellulose, and to evaluate the performance of SBA-15/cellulose loaded with antibiotics for potential wound dressing application. The morphology, topography, structure and porosity of composite membranes and SBA-15 mesoporous particles were examined by SEM, TEM and BET. Tensile strength, water vapor transmission rate (WVTR), swelling properties, drug release profile and the antibacterial properties against *Staphylococcus aureus* and *Eschericia coli* were studied. Overall, SBA-15/cellulose composite demonstrated a sustained release profile of antibiotics. Interestingly, the results supported the protection mechanism of CaCO_3_ for SBA-15 during the environmentally benign synthesis of composite membrane under alkaline condition.

## 2. Materials and Experimental Methods

### 2.1. Materials

Cellulose was kindly provided by Hubei Golden Ring Co., Ltd. (Xiangyang, China). Its viscosity–average molecular weight (*M*_η_) was 9.5 × 10^4^ Da, as calculated with a Mark–Houwink equation after obtaining the measurement from an Ubbelohde viscometer (Mesu Lab Instruments (Guangzhou) Co., Ltd., Guangzhou, China) at 25 ± 0.05 °C under LiOH/urea aqueous solution. Poly(ethylene glycol)-block-poly(propylene glycol)-block-poly(ethylene glycol) (P123, average M_n_ ~5800) was purchased from Sigma Aldrich (St. Louis, MO, USA). Chloramphenicol (purity > 98%) was obtained from Aladdin. Sodium hydroxide (NaOH), urea, hydrochloric acid, sulfuric acid, ethylsilicate (TEOS), calcium chloride (CaCl_2_), sodium carbonate (Na_2_CO_3_) and other chemicals were supplied by Sinopharm Chemical Reagent Co., Ltd (Shanghai, China), and were used without further purification. Phosphate-buffered saline (PBS) was prepared in our laboratory.

### 2.2. Preparation of Mesoporous SBA-15 Particles and CaCO_3_-Protected SBA-15

SBA-15 microparticles were prepared by a published protocol with a slight modification [33]. In brief, SBA-15 mesoporous molecular sieves were produced by hydrothermal crystallization in an acidic environment using nonionic surfactant P123 as template, and tetraethyl orthosilicate (TEOS) as the silicon source. Firstly, 4 g of P123 was added into a round-bottomed flask containing 150 mL of 1.6 M HCl aqueous solution, then stirred at 35 °C for 24 h. Afterwards, 8.5 g of TEOS was rapidly added to the above solution under vigorous stirring. After stirring for 5 min, the mixture was settled at 35 °C for 20 h, then transferred to a Teflon-lined autoclave and heated at 130 °C for 24 h. After filtering, washing with water/ethanol solution, drying, and calcining at 550 °C for 6 h, SBA-15 was obtained. To prepare CaCO_3_-protected SBA-15, a given amount of SBA-15 was dispersed in 10 wt% CaCl_2_ solution with a mass ratio of SBA-15 particles to CaCl_2_ set at 1:1, 1:2 or 1:3, followed by soaking for 48 h. Afterwards, SBA-15 suspension at a particular SBA-15:CaCl_2_ ratio was mixed with 10 wt% Na_2_CO_3_ solution and mechanically stirred for 2 h. After repeatedly washing with deionized water and drying in an oven, CaCO_3_-coated SBA-15 particles were obtained. CaCO_3_-coated SBA-15 particles with SBA-15:CaCl_2_ ratios of 1:1, 1:2 and 1:3 were denoted as Ca1-SBA, Ca2-SBA and Ca3-SBA, respectively.

### 2.3. Preparation of SBA-15/Cellulose Composite Membrane

#### 2.3.1. Using CaCO_3_-Protected SBA-15

**Scheme 1:**
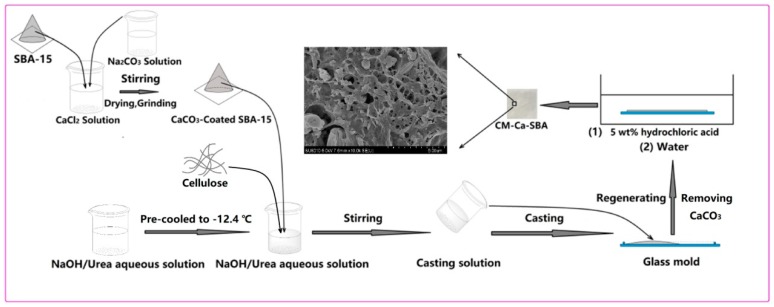
Diagrammatic approach to the preparation of CaCO3-protected SBA-15/cellulose composite membrane (CM-Ca-SBA).

As shown in Scheme 1, a weighed content of CaCO_3_-coated SBA-15 (Ca1-SBA, Ca2-SBA, Ca3-SBA) particles was added into 200 g of working solution (7 wt% NaOH, 12 wt% urea, 81 wt% deionized water) at −12.4 °C. Next, 8 g of cellulose was added and stirred for 5 min to obtain a casting solution containing different weight contents of CaCO_3_-coated SBA-15 particles. The prepared casting solution was poured into a glass mold with a thickness of 0.5 mm and immersed into 5 wt% hydrochloric acid solution for 24 h to remove CaCO_3_ layer of SBA-15. During this process, CaCO_3_ was totally converted to calcium chloride and carbon dioxide, which was confirmed by the absence of further gas bubble generation. After washing with deionized water five times, the as-obtained SBA-15/cellulose membranes with different weight contents of SBA-15 at 10 wt%, 20 wt% and 30 wt% were denoted as CM-SBA(10%), CM-SBA(20%) and CM-SBA(30%), respectively. Ca1-SBA, Ca2-SBA and Ca3-SBA were employed to fabricate SBA-15/cellulose membranes, which were designated as CM-Ca1-SBA, CM-Ca2-SBA and CM-Ca3-SBA, respectively. For example, SBA-15/cellulose membrane with 30 wt% of SBA-15 (Ca2-SBA) was denoted as CM-Ca2-SBA(30%).

#### 2.3.2. Using Unprotected SBA-15

A given amount of SBA-15 particles were dispersed in 200 g pre-prepared cellulose solution cooled to –12.4 °C. Afterwards, 8 g cellulose was added immediately and stirred for 5 min to obtain a casting solution. The casting solution as poured into a glass mold with a thickness of 0.5 mm and then incubated in 5 wt% hydrochloric acid solution for 24 h to obtain 30 wt% unprotected SBA-15/cellulose composite membranes, denoted as CM-U-SBA. To investigate the effect of cellulose casting solution on the pore structure of SBA-15-based particles, CM-U-SBA(30%), CM-Ca1-SBA(30%), CM-Ca2-SBA(30%) and CM-Ca3-SBA(30%) were calcined by air in a muffle furnace at 550 °C.

### 2.4. Characterization

Field emission scanning electron microscopy (SEM, Hitachi SU8010, Tokyo, Japan) with an acceleration voltage of 5 KV was applied to probe the microstructures of SBA-15-based particles and SBA-15/cellulose composite membranes. Transmission electron microscopy (TEM, Tecnai G2 20 S-TWIN, FEI Company, Hillsboro, OR, USA) was conducted under an acceleration voltage of 200 KV to examine the microstructures of pristine SBA-15 and calcined CM-Ca2-SBA(30%).

To examine the pore structure of SBA-15-based nanoparticles and SBA-15/cellulose composite membranes, nitrogen adsorption–desorption isotherms at 77 K were acquired by a computer-controlled ASAP 2010 physisorption apparatus (Micromeritics Instrument Corp., Norcross, GA, USA). The specific surface area of synthesized SBA-15, calcined CM-U-SBA, calcined CM-Ca1-SBA(30%), calcined CM-Ca2-SBA(30%) and calcined CM-Ca3-SBA(30%) were obtained by Brunauer–Emmett–Teller analysis (BET) [34], while mesoscale pore size distribution and mesopore volume were achieved by the BJH method [35]. The chemical structure of SBA-15-based particles and SBA-15/cellulose composite membranes were measured by Fourier transform infrared spectroscopy (FTIR, Nicolet 5700, Thermo Fisher Scientific Inc., Waltham, MA, USA). Their crystal structure was characterized by X-ray diffraction (XRD, Bruker D8 Advance, Bruker Corporation, Billerica, MA, USA) using Cu Kα radiation (λ = 1.5406 Å). The θ scan data were collected from 5.0° to 80.0° under a scanning speed of 2.0°/min.

### 2.5. Mechanical Properties Evaluation

A CMT8202 universal mechanical testing machine (MTS Systems Corporation, Eden Prairie, MN, USA) using a 200-N load cell [36,37] was applied to investigate the mechanical properties of various membranes. Rectangular specimens were cut into 60 mm × 5 mm and extended at a constant crosshead speed of 20 mm/min with a 40-mm gauge length. Each type of sample was tested 10 times to determine the mean value.

### 2.6. Swelling Behavior Test

On the basis of the gravimetric method [38,39], the swelling behavior of various types of membranes were assessed in PBS medium. The specimens were cut into 2 mm × 2 cm squares, weighed (*W*_0_) and then soaked in 10 mL of 0.1 M PBS (pH 7.4) at 37 °C for 24 h. Subsequently, the swollen samples were taken out from the solution, lightly wiped off with filter paper to clean the surface solution and immediately weighed (*W_t_*). The swelling ratio was calculated with the following equation:

Swelling ratio (%) = (*W_t_*−*W*_0_)/*W*_0_ × 100 (1)

### 2.7. Moisture Permeability Test

The water vapor transmission rate (WVTR) of the specimens was tested according to the ASTM E96-00 method [39]. During the test, each membrane sample was utilized to seal the opening of a cylindrical cup containing 10 mL deionized water and prevent vapor escaping from the rim. The cups were then placed in an oven containing (NH_4_)_2_SO_4_ saturated solution at 37 °C to maintain 75% relative humidity. A totally open plastic cup was taken as the blank control. At different time points, the cups were weighted and the WVTR was determined by the following equation:

(2) $$\mathrm{WVTR}\;(g\cdot m^{-2}\cdot {\mathrm{day}}^{-1})=\frac{\frac{\Delta m}{\Delta t}}{A},$$

where Δm/Δt is the weight of moisture loss for 24 h (g/day), and *A* is the membrane area exposed to the moisture transfer (m^2^).

### 2.8. Drug Loading and Releasing Experiment

The cellulose and SBA-15/cellulose membranes were immersed in a chloramphenicol–ethanol solution for 48 h in order to load the drugs into the membrane matrix. The concentration of chloramphenicol in solution was determined by an ultraviolet spectrophotometer (Spectra Max M2e, Molecular Devices Company, Sunnyvale, CA, USA), and drug loading was calculated based on the concentration difference before and after absorption. The membranes that absorbed chloramphenicol were washed twice with anhydrous ethanol to remove the weakly bounded drugs, swapped with filter papers and dried in the vacuum. During the drug release test, 0.15 g drug-loaded SBA-15/cellulose membranes were placed in 20 mL PBS solution. At certain time intervals, 1-mL samples were withdrawn from the above solution to measure the UV absorbance, and then replenished with fresh PBS. The drug releasing rate was determined in terms of the following formula:

(3) $$\mathrm{Drug}\;\mathrm{release}\;(\%)\;=\;\frac{\mathrm{amount}\;\mathrm{of}\;\mathrm{released}\;\mathrm{drug}}{\mathrm{amount}\;\mathrm{of}\;\mathrm{loaded}\;\mathrm{drug}}\;\times 100,$$

### 2.9. Antibacterial Activity

*S. aureus* (ATCC 25923) and *E. coli* (ATCC 25922) were used to assess the antibacterial activity of the composite membranes [40,41]. A 6.0-mm diameter disc was punched out of the membrane with a perforator. The solidified agar was prepared by dissolving 0.5 g beef soup, 1 g agar, 0.5 g NaCl and 1.5 g peptone in 100 mL distilled water, and was evenly coated on the culture dish. Afterwards, the bacteria (10^6^ CFU/mL) were evenly spread onto the surface of the solidified agar and the circular specimens of composite membrane were placed in the solidified agar to inoculate with tested bacteria. Bacteria culture sat for 144 h at 37 °C, then the diameter of the inhibition zones was measured at several predetermined time points. Each test was repeated three times.

### 2.10. Statistical Analysis

Each test was repeated five times unless otherwise specified, and the data are shown as mean ± standard deviation. A one-way ANOVA analysis was performed to compare the mean values among different groups. Probability values of *p* < 0.05 were considered statistically significant.

## 3. Results and Discussions

### 3.1. The Protect Effect of CaCO_3_ on SBA-15 in Cellulose Solvent

**Figure 1:**
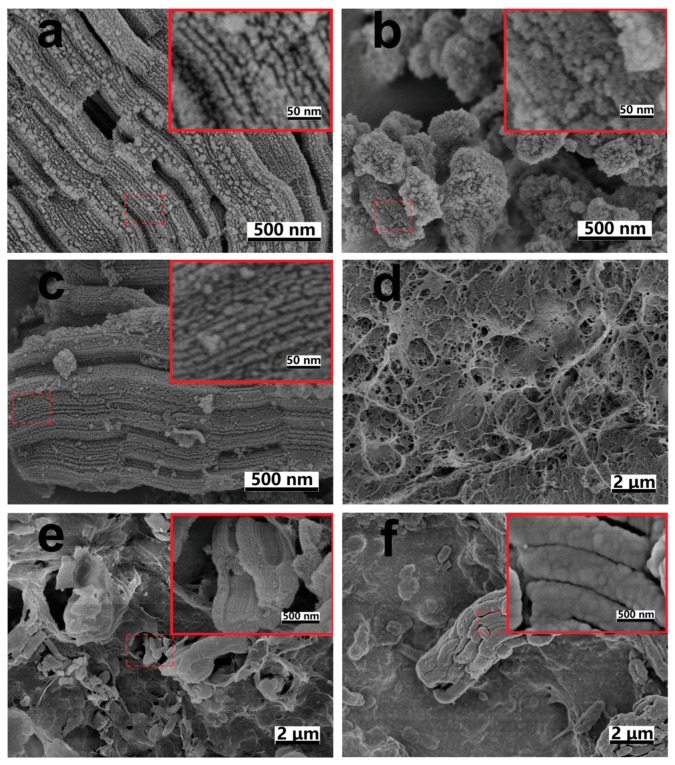
SEM images of (**a**) pristine SBA-15 particles; (**b**) CaCO_3_-coated SBA-15 particles, (**c**) remnant of calcined CM-Ca2-SBA(30%), (**d**) CM, (**e**) CM-Ca2-SBA(30%) and (**f**) CM-U-SBA(30%).

To verify the protective effect of CaCO_3_ coating on the pore structure of SBA-15 microparticle, the morphology of SBA-15-based particles with or without CaCO_3_ coating was probed. SEM images of pristine SBA-15 particles, CaCO_3_-coated SBA-15 particles, calcined CM-Ca2-SBA(30%), plain CM, CM-Ca2-SBA(30%) and CM-U-SBA(30%) are shown in Figure 1. It can be clearly seen that pristine SBA-15 particles displayed a stripe-patterned channel composed of open pore walls and gullies (Figure 1a). However, the morphology as mentioned above could hardly be found on the surface of SBA-15 particles after coating with a layer of CaCO_3_ (Figure 1b). During the preparation of CaCO_3_-coated SBA-15, the interfacial reaction of CaCl_2_ ions absorbed on the outer surface of SBA-15 with Na_2_CO_3_ ions in solution formed a layer of CaCO_3_ coating outside the SBA-15. In addition, certain suspended CaCO_3_ particulates formed in the reaction mixture might have been deposited onto SBA-15 surface, partially contributing to the formation of the CaCO_3_ protective layer that filled up the stripe-like channel in pristine SBA-15. As shown in Figure 1d,e, CM-Ca2-SBA(30%) presented the characteristic micro- and nano-porous network of pure cellulose membrane. By comparing Figure 1a to Figure 1c, CaCO_3_-protected SBA-15 particles incorporated within CM-Ca2-SBA(30%) displayed the typical morphology of the pristine SBA-15 particles after the calcination of the composite gel (removal of cellulose). Moreover, the stripe-patterned channel of pristine SBA-15 is clearly shown within the matrix of CM-Ca2-SBA(30%) (inset, Figure 1e). In contrast, the characteristic morphology of the SBA-15 particle was not found in CM-U-SBA(30%) (inset, Figure 1f) when CaCO_3_ coating was not applied on SBA-15 before membrane casting. The result strongly indicates that NaOH used in membrane casting reacted with the pristine silica microparticle, leading to an eruption of the morphological features, while the CaCO_3_ layer protected the silica against NaOH etching. Eventually, the CaCO_3_ layer was removed by HCl after the casting step and the pristine pore structure of SBA-15 was restored. 

**Figure 2:**
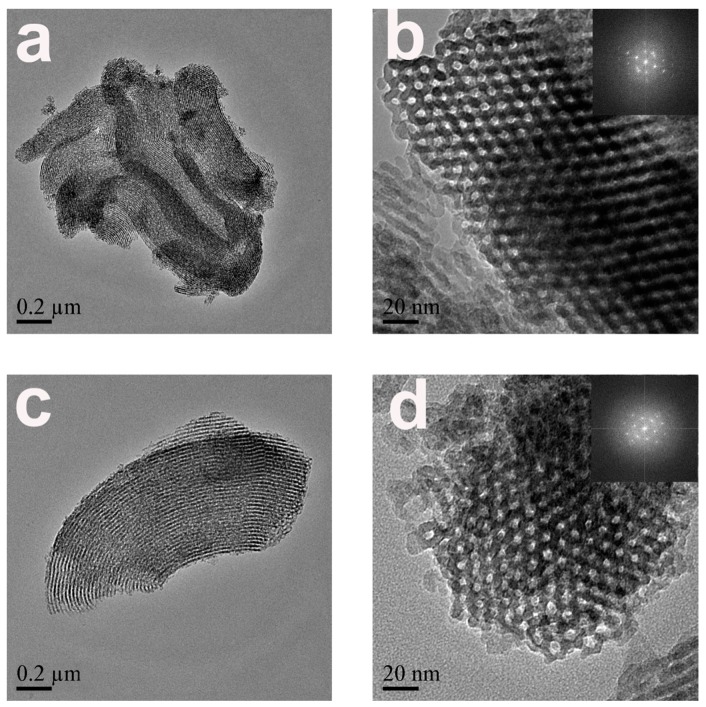
TEM images of (**a**,**b**) pristine SBA-15 and (**c**,**d**) remnant of calcined CM-Ca2-SBA(30%).

TEM was applied to probe the nanoscale morphology and structure of SBA-15 particles. Pristine SBA-15 was comprised of monodispersed rodlike particles of 0.05~0.2 µm in diameter and 0.5~1.5 µm in length. TEM images of pristine SBA-15 and calcined CM-Ca2-SBA(30%) displayed that the stripes were parallel to, and pore channels were perpendicular to, the pore axis (Figure 2a–d). The stripe parallel to the pore channels (110) can be clearly seen in Figure 2a,c, confirming the protective effect of CaCO_3_ in reducing the chemical etching of silica in an alkali environment, which is consistent with the SEM results. As shown in Figure 2b,d, both pristine SBA-15 and calcined CM-Ca2-SBA(30%) displayed highly ordered two-dimensional hexagonal pore channels perpendicular to the pore axis (100), supporting the notion that the CaCO_3_ layer can also protect the surface of the inner channel of SBA-15, and not just the outer surface. 

To determine the protective effect of calcium carbonate on the SBA-15 pore structure, a BET test was also performed. Figure S1a–e (Supplementary Materials) shows the BET adsorption isotherms and BJH pore distribution curves of pristine SBA-15 particles, calcined CM-U-SBA(30%), calcined CM-Ca1-SBA(30%), calcined CM-Ca2-SBA(30%) and calcined CM-Ca3-SBA(30%). Table 1 summarizes the BET and BJH results of the five types of particles as mentioned above. As listed in Table 1, SBA-15 without any protection (calcined CM-U-SBA(30%)) displayed a significant reduction in the specific surface area (from 494 ± 27.5 to 174 ± 45.0 m²/g), pore size (from 8.96 ± 0.19 nm to 13.6 ± 2.22 nm) and pore volume (from 1.31 ± 0.07 cm^3^/g to 0.71 ± 0.12 cm^3^/g) compared with those of the pristine SBA-15 (n = 5, *P* < 0.05). The results mentioned above strongly imply that the mesoporous structure of SBA-15 did not withstand the strong alkaline environment in the absence of protection strategies, as OH^-^ ions entered the pores of the cellulose matrix and reacted with silica during the preparation of composite membrane. As a result, the chemical etching damaged the pore structure through partial erosion of the channel wall. In addition, a large number of pores expanded, inducing a dramatic reduction of pore volume and specific surface area. For calcined CM-Ca1-SBA(30%), there was still certain decrease of surface area (from 494 ± 27.5 to 379 ± 43.7 m²/g) and pore volume (from 1.31 ± 0.07 cm^3^/g to 1.06 ± 0.08 cm^3^/g) compared with those of the pristine SBA-15 (n = 5, *P* < 0.05), but the extent of change was significantly less than those of the CM-U-SBA(30%) and pristine SBA-15. Therefore, the incomplete CaCO_3_ layer offers limited protection activity to SBA-15 during the preparation of SBA-15/cellulose composite membrane. With the use of Ca2-SBA-15 and Ca3-SBA-15, calcinated CM-Ca2-SBA(30%), CM-Ca3-SBA(30%) and pristine SBA-15 particles possess similar values of specific surface area, pore size and pore volume (n = 5, *P* > 0.05) (see Table 1). The results further support that a sufficient amount of precoated CaCO_3_ layer on SBA-15 is critical for preserving the basic integrity of the pore channel during alkaline processing and the acidic recovery of cellulose composite membrane. 

### 3.2. Structure of Composite Membrane 

**Figure 3:**
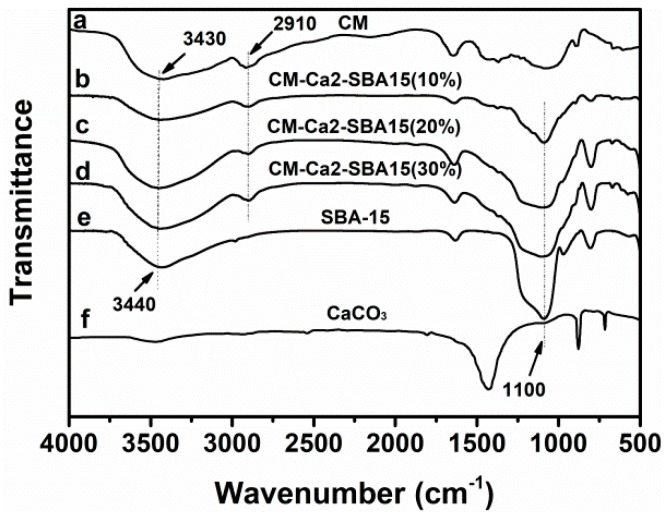
FTIR spectra of (**a**) CM, (**b**) CM-Ca2-SBA(10%), (**c**) CM-Ca2-SBA(20%), (**d**) CM-Ca2-SBA(30%), (**e**) SBA-15 and (**f**) CaCO_3__._

FTIR measurements were applied to probe the interactions between SBA-15 and cellulose. The FTIR spectra of pure CM, CM-Ca2-SBA(10%), CM-Ca2-SBA(20%), CM-Ca2-SBA(30%), pristine SBA-15 particles and CaCO_3_ are shown in Figure 3. CM shows a strong absorption peak (3440 cm^−1^) and an OH– and –CH stretching vibration peak (2910 cm^−1^), which is consistent with other published results [42]. In SBA-15/CM-based composite membranes, the peak at 1110 cm^−1^ appears in addition to the characteristic peaks of celluloses, which were attributed to the Si–O–Si network of the SBA-15 particle [32]. It is noteworthy that the characteristic peaks of CaCO_3_ were not detected in composite membrane (Figure 3g), implying that CaCO_3_ coating of SBA-15 surface was effectively removed from the membrane after hydrochloric acid treatment and subsequent washing steps.

The wide-angle diffraction (XRD) patterns of pure CM, CM-Ca2-SBA(10%), CM-Ca2-SBA(20%), CM-Ca2-SBA(30%) and pristine SBA-15 particles are shown in Figure S2 (Supplementary Materials). CM displays diffraction peaks at diffraction angles of 12.2, 19.9 and 21.1 degrees, corresponding to (110), (220) and (200) planes, respectively, which are characteristic peaks of type II cellulose [43]. There was a negligible crystallization peak in pure SBA-15 powder, because the silica skeleton of SBA-15 is amorphous. As shown in Figure S2b–d, SBA-15/cellulose composite membranes display three characteristic peaks of type II cellulose, but the intensity of those peaks shows a decreasing trend with the increase of SBA-15 content and the corresponding reduction of cellulose content. It is noteworthy that no crystallization peak of CaCO_3_ is observed on the composite membrane, confirming that the CaCO_3_ were removed completely through HCl treatment.

### 3.3. Effects of Hydrochloric Acid Treatment and Addition of SBA-15 on Mechanical Properties

**Figure 4:**
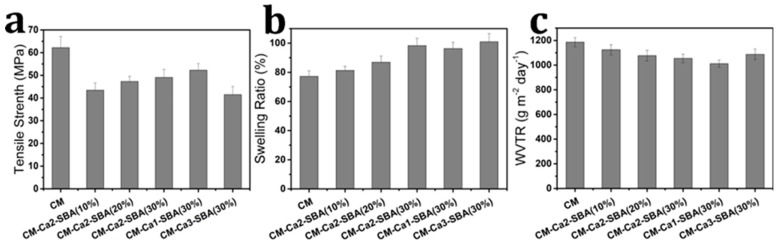
(**a**) Tensile strength, (**b**) swelling ratio and (**c**) water vapor transmission rate (WVTR) of each sample.

Wound dressings should provide good mechanical properties in order to protect the wound bed from further injury [36,44]. Figure 4a shows the effect of the SBA-15 particle contents on the tensile strength of the composite membranes. The average tensile strength of CM, CM-Ca2-SBA(10%), CM-Ca2-SBA(20%) and CM-Ca2-SBA(30%) were 62.2, 43.5, 47.3 and 49.1 MPa, respectively. Generally, the addition of hard nanofillers to cellulose may enhance its mechanical properties. In our study, lower tensile strengths of the composite membranes compared with those of pure cellulose membrane may have resulted from the treatment with hydrochloric acid during the preparation of SBA-15/cellulose composite membranes. HCl is likely to induce partial hydrolysis of cellulose and damage the integrity of membrane, resulting in an impaired mechanical performance. However, the tensile strengths of composite membranes still increased with the increase of SBA-15 content. Similar enhancement effects of nanoparticles on cellulose membranes have been found in other reports [45]. At present, the tensile strength of widely used skin tissue repair materials, such as Intrgra, Pelnac, Biofill and Gengiflex, is around 0.48 MPa or even lower [46]. SBA-15/cellulose composite membranes show higher tensile strength than that of the aforementioned commercial products, which is favorable for wound healing.

### 3.4. Effect of Adding SBA-15 on Swelling Behavior

In order to absorb body fluids and prevent excessive humoral secretions from accumulating in the wound bed, the wound dressing should provide good swelling properties [44]. The swelling ratio is used to characterize the fluid absorption capacity. The swelling ratio of freeze-dried CM and composite membranes in phosphate buffer (PBS, pH 7.4) are shown in Figure 4b. The swelling rates of CM, CM-Ca2-SBA(10%), CM-Ca2-SBA(20%) and CM-Ca2-SBA(30%) are 77.2%, 81.3%, 86.9% and 98.3%, respectively. It is obvious that the addition of SBA-15 to cellulose membrane induces the increase of the swelling capacity to some extent, which may be closely related to the high porosity and strong water absorption of SBA-15. Thus, the addition of mesoporous silica in membranes is beneficial for absorbing body fluid.

### 3.5. Water Vapor Transmission Rate

During the course of wound healing, whether or not the wound dressing provides a moist environment required for wound healing is important, which is closely related to the moisture permeability of membrane materials [44]. The wound dressing should not only prevent the wound from drying and producing scar due to an excessive WVTR, but should also avoid the accumulation of exudates, which lead to bacterial growth and delay the wound healing process owing to too low of a WVTR [47]. The water vapor permeability of pure CM and composite membrane (0.05 mm in thickness) are shown in Figure 4c. The water vapor permeability of CM, CM-Ca2-SBA(10%), CM-Ca2-SBA(20%) and CM-Ca2-SBA(30%) are 1186, 1125, 1077 and 1054 g·m^−2^·d^−1^, respectively. The moisture permeability of wound repair materials, such as membrane material, should lies between 904 and 1447 g·m^−2^·d^−1^ for clinical application [44]. It can be seen that the moisture permeability of the composite membranes ranged from 1012 to 1186 g·m^−2^·d^−1^, meeting the requirement of clinical application.

### 3.6. Drug Loading and Release 

**Figure 5:**
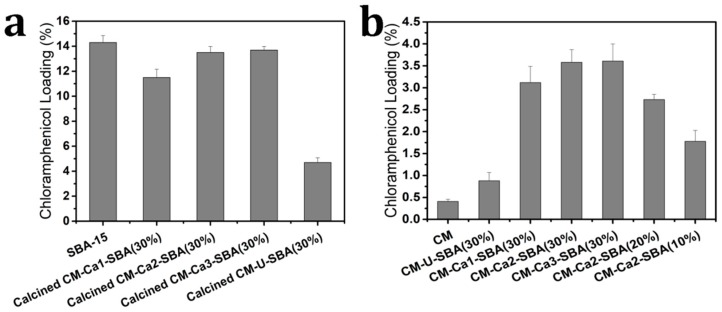
Chloramphenicol loading percentage of (**a**) each particle, and (**b**) dry membrane samples.

Antibacterial activity is closely related to drug loading capacity of the dressing. Chloramphenicol, which is a typical inflammatory or anti-infective drug, was loaded into the composite membrane for the functionalization of wound dressing herein. As shown in Figure 5a, the amount of chloramphenicol loading in pristine SBA-15 particles, calcined CM-Ca1-SBA(30%) particles, calcined CM-Ca2-SBA(30%) particles, calcined CM-Ca1-SBA(30%) and calcined CM-U-SBA(30%) particles was 14.3%, 11.5%, 13.5%, 13.7% and 4.7%, respectively. Apparently, the drug loading capacity of pristine SBA-15 particles was the highest among all samples. The calcined CM-Ca2-SBA(30%) particles and calcined CM-Ca3-SBA(30%) possessed a comparable loading capacity to pristine SBA-15 due to an almost-intact mesoporous structure, affirming the effectiveness of the CaCO_3_ protection strategy in the porous network of silica particles. Interestingly, the drug loading capacity of calcined CM-Ca1-SBA(30%) displayed a slight reduction from 14.3% (pristine SBA-15) to 11.5% compared to the pristine SBA-15, indicating a limited protection activity owing to insufficient CaCO_3_. When no protection strategy was used in the preparation of SBA-15/cellulose membranes, the amount of drug loaded dropped sharply to 4.7%, owing to the seriously damaged mesoporous structures of silica particles. The results of loading capacity are in accordance with SEM, TEM and BET results. The loading amount of CM, CM-U-SBA(30%), CM-Ca1-SBA(30%), CM-Ca2-SBA(30%), CM-Ca3-SBA(30%), CM-Ca2-SBA(20%) and CM-Ca2-SBA(10%) membranes are shown in Figure 5b, corresponding to the results of 0.4%, 0.9%, 3.1%, 3.6%, 3.7%, 2.7% and 1.8%, respectively. Despite the damaged mesoporous structure of SBA-15, the amount of drug loaded in CM-U-SBA(30%) was 125% higher than that of CM. With the limited protection effect due to insufficient CaCO_3_ coating, the amount of drug loaded in CM-Ca1-SBA(30%) increased to 3.1%, which was 244% higher than that of CM-U-SBA(30%). The amount of drug loaded in CM-Ca2-SBA(30%) and CM-Ca3-SBA(30%) were highest of the seven specimens studied herein, indicating the most effective protection brought by a sufficient amount of CaCO_3_. The significant difference of drug-loading capacity between CM and protected SBA-15/CM suggests that most of the drug may stay inside the mesoporous structure of SBA-15. In addition, a higher weight content of CaCO_3_-coated SBA-15 suggests larger specific surface area allowing for drug adsorption, which is consistent with the sequence of loaded drug amounts (CM-Ca2-SBA(30%) > CM-Ca2-SBA(20%) > CM-Ca2-SBA(10%)). 

**Figure 6:**
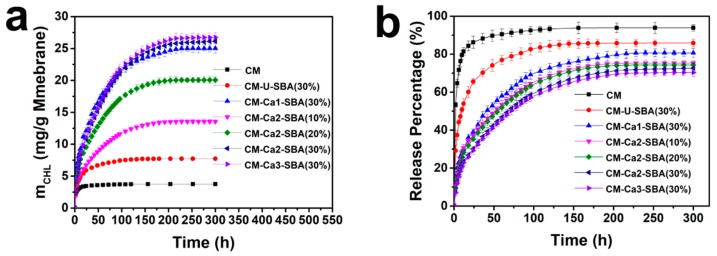
(**a**) Cumulative release mass and (**b**) release profile of chloramphenicol for different CM-based membranes in PBS.

Sustained drug release is desirable for wound dressing in order to provide long-term antibacterial activity for wound healing. Figure 6a,b shows the release amount and release percentage of chloramphenicol of CM, CM-U-SBA(30%), CM-Ca1-SBA(30%), CM-Ca2-SBA(30%), CM-Ca3-SBA(30%), CM-Ca2-SBA(20%) and CM-Ca1-SBA(30%) in PBS (0.1 M, pH 7.4). As shown in Figure 6a, the mass of drug release from the composite membrane was higher than that of CM, which is attributed to the significantly higher amount of loaded drug inside composite membranes than CM, despite of the lower drug release percentage. CM displayed a burst release profile during the initial 12 h, and then reached its peak value. The half release time of CM was only 2 h. After 24 h, drug release completely ceased. Owing to the structural characteristics of cellulose, it was convenient for drugs confined in the pore of cellulose membrane to release through simple diffusion and reach the external microenvironment, producing the rapid and short-time release behavior. With the inclusion of SBA-15 particles, the initial drug release rates of CM-Ca1-SBA(30%), CM-Ca2-SBA(30%) and CM-Ca3-SBA(30%) were significantly lower than that of CM. Furthermore, sustained drug release of the composite membrane can last for 150 h. The better preservation of the SBA-15 mesoporous structure led to the longer drug release duration, as shown in the composite membranes. Furthermore, the higher the loading amount of CaCO_3_-coated SBA-15 gets, the longer it prolongs the drug release at a steady rate. In particular, CM-Ca2-SBA(30%) presented a slow and constant release behavior for nearly 160 h after the initial 24 h of rapid release, and the drug was released at a rate of 2.3 mg/day per unit mass of the membrane.

As mentioned above, the majority of drug loaded within SBA-15/CM membranes was located inside the mesopore of SBA-15. Thus, the drugs had to diffuse out of the mesopores into the cellulose’s micro-/nano-scale pore channel, then escape from the pore of cellulose membrane to the external microenvironment. The abrupt drug release profile resulted from the first stage of diffusion, if any, would be restricted by the second stage of diffusion cascade. Therefore, the two-stage release activities endorsed the sustained drug release at a stable rate for a longer time range. Compared with MCM-41/CMC hydrogel [32,45], the duration of the sustained drug release of CM-Ca2-SBA(30%) increased from 70 to 270 h. The drug release extent of these composite membranes could not reach 100%. Especially the release rate of CM-Ca2-SBA(30%) only reached 69%. It was possible that some drugs interacted with SBA-15 through the formation of hydrogen bonds, which prevented the effective release of strongly bound drug molecules. After swelling of the membrane, some mesoporous pore channels of SBA-15 were likely to be blocked by a macromolecular chain of cellulose, and thus drugs in mesoporous pores were difficult to release. Due to the almost-intact mesoporous structure of SBA-15, CM-Ca2-SBA(30%) and CM-Ca3-SBA(30%), applying a CaCO_3_ protection strategy demonstrated sustained release behavior, which could potentially exert a prolonged antibacterial effect on the wound bed. It should be noted that CM-Ca2-SBA(30%) and CM-Ca3-SBA(30%) had comparable drug loading capacities and sustained release profiles, but CM-Ca2-SBA(30%) possessed better mechanical properties than those of CM-Ca3-SBA(30%). Though the initial abrupt drug release still existed for CM-Ca2-SBA(30%), this characteristic is desirable in the treatment of skin injury, and therefore CM-Ca2-SBA(30%) should be chosen as the potential candidate for wound dressing development, as it possesses excellent mechanical properties and provides the optimum antibacterial protection for the wound. 

### 3.7. Drug Release Mechanism 

There are three possible drug release mechanisms from hydrogel that can be used as a drug delivery system: drug diffusion, hydrogel swelling and hydrogel erosion or dissolution. According to the published results, drug release from mesoporous silica particles including SBA-15 is usually a diffusion process [48]. Drug release from cellulose membranes also proceeds with a diffusion mechanism [49]. Therefore, the release of chloramphenicol from SBA-15/cellulose composite membranes may be considered to be a diffusion process. To verify the aforementioned assumption and gain a better understanding of the kinetics and drug release mechanism of these composite membranes, experimental drug release data were analyzed by a power law model [50]. The power law model, Equation (4), is the result of solving the diffusion equation in the early drug release time scale, which holds for up to 60% of the release:

(4) $$\frac{M_{t}}{M_{\infty }}=Kt^{n},$$

where the *M_t_/M*_∞_ is a fraction of the drug released at time *t,* and *K* is the kinetic constant depending on the penetration coefficient of the drug in related matrices, incorporating the structure and geometric characteristics of the drug delivery system, and *n* is the diffusion exponent revealing the drug release mechanism from the polymeric matrices. The drug release diffusion control region profile has been considered, and the values of *K* and *n* were determined by fitting the drug release data into the power law equation, as shown in Table S1 (Supplementary Materials). From the kinetics model analysis herein, *n* < 0.45 reveals that the drug release from the SBA-15/cellulose composite membrane belongs to Fick diffusion, consistent with the innate porous structure of CM and mesoporous SBA-15 particles. In fact, the Fick diffusion-controlled process is common in drug-loaded particle/polymer composites such as mesoporous silica nanoparticles (MSNs)/chitosan and iron oxide nanoparticles/PVA composites [51,52].

### 3.8. Antibacterial Assessment

**Figure 7:**
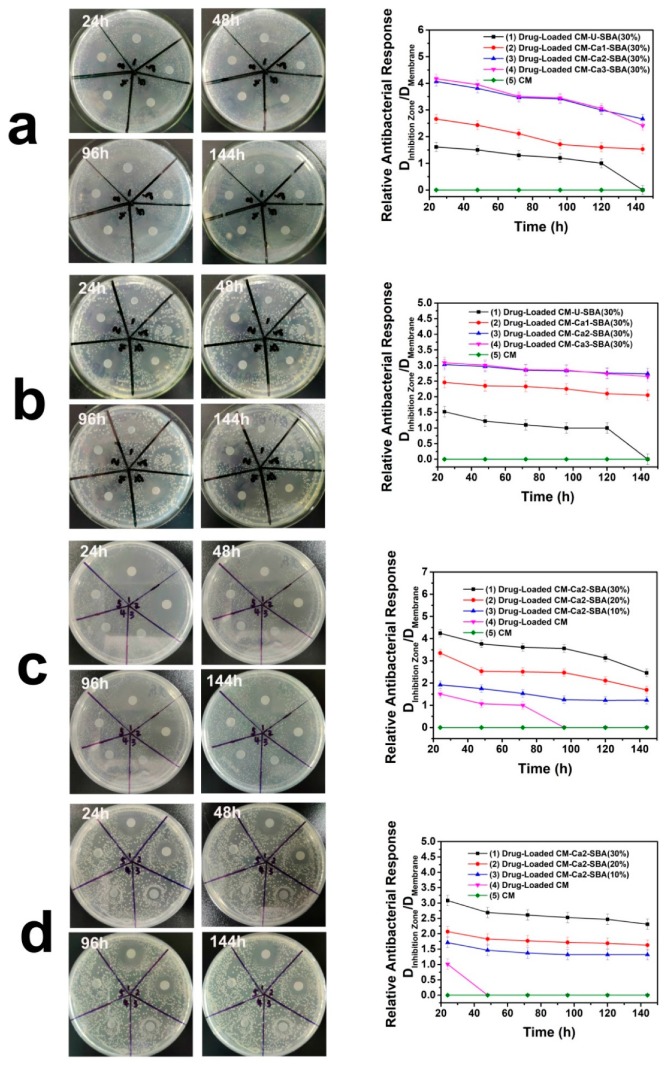
Photograph and the relative antibacterial response of chloramphenicol-loaded plain CM and CM-based membranes against (**a**,**c**) *S. aureus* and (**b**,**d**) *E.coli*.

The chloramphenicol-loaded plain CM and CaCO_3_-coated SBA-15/CM membranes were assayed for their long-term antibacterial activity against *S. aureus* and *E. coli*. As shown in Figure 7a,b, the ratio of the inhibition zone diameter to the membrane diameter for the various types of cellulose-based composite membranes was in the decreasing order as follows: drug-loaded CM-Ca3-SBA(30%) = drug-loaded CM-Ca2-SBA(30%) > drug-loaded CM-Ca1-SBA(30%) > drug-loaded CM > plain CM. Although the weight content of SBA-15 in the three composite membranes was identical, CM-Ca3-SBA(30%) and CM-Ca2-SBA(30%) showed the largest size of inhibition zone for both types of bacteria, which is just consistent with the results of the cumulative release mass and the release profile in Figure 7. The effect of the extra amount of SBA-15 or chloramphenicol on the size of the inhibition zone is shown in Figure 7c,d. With the increase of SBA-15 content, the size of the inhibition zone increases as expected in a given time period. Interestingly, all three SBA-15/cellulose samples with loaded drugs had strong antibacterial ability against both of *S. aureus* and *E. coli* for 144 h, which is complementary to the recovery of injury. In comparison, due to the low drug-loading capacity and rapid initial release, the shrinkage of the inhibition zone on CM proceeded quite rapidly, reflecting the limited antibacterial activity of the plain drug-loaded CM. Therefore, the inclusion of CaCO_3_ protected SBA-15 (30%) is an effective strategy for the preparation of antibacterial CM-based membranes for wound dressing application. It should be pointed out that CM-Ca2-SBA(30%) demonstrated the highest antibacterial activity in terms of sustained release time, and the medium relative antibacterial response when compared with other drug release systems previously reported (Table 2), confirming the promise of our strategy in controlling the drug release of CM with CaCO_3_-protected SBA-15. 

## 4. Conclusions

In this study, a CaCO_3_ layer was effectively immobilized on the internal pores and outer surface of mesoporous SBA-15 to prevent NaOH from etching the SBA-15 surface, which was verified by the preservation of mesoporous morphology of SBA-15 before and after cellulose composite preparation. The cellulose-based composite membranes with hierarchical porous structure were prepared successfully by the incorporation of CaCO_3_-coated SBA-15 into cellulose membranes. The high porosity and specific surface area of SBA-15 and the innate nano/micro pore of cellulose membrane endowed SBA-15/cellulose membranes with a high drug-loading capacity. After loading chloramphenicol, cellulose-based composite membranes exhibited a moderate amount of initial rapid release and a subsequent slow and constant release profile. In particular, the drug release of composite membranes containing 20% and 30% SBA-15 maintained a stable state within 170 h after the initial rapid release phase, then proceeded with a slow release process up to 270 h, which achieved the goal of a slow and sustained release. This membrane developed herein was in accordance with the recovery requirements of skin injury. Most importantly, the composite membrane exhibited persistent antibacterial ability over 144 h in light of the extensive inhibition zone. Furthermore, the mechanical property, swelling behavior and water vapor transmission rate were evaluated, complying with the primary requirements for wound dressing materials. All these results indicated the potential of cellulose-based composite membranes for application as antimicrobial dressing for wound healing.

## Figures and Tables

**Table 1:** The specific surface area, pore diameter and pore volume analysis of particles.

Sample	Surface (m²/g)	Pore Size (nm)	Pore Volume (cm^3^/g)
Pristine SBA-15	494 ± 27.5	8.96 ± 0.19	1.31 ± 0.07
Calcined CM-Ca3-SBA(30%)	487 ± 9.70	9.08 ± 0.32	1.29 ± 0.02
Calcined CM-Ca2-SBA(30%)	482 ± 9.73	9.07 ± 0.04	1.30 ± 0.06
Calcined CM-Ca1-SBA(30%)	379 ± 43.7	9.23 ± 0.35	1.06 ± 0.08
Calcined CM-U-SBA(30%)	174 ± 45.0	13.6 ± 2.22	0.71 ± 0.12

**Table 2:** Comparison of drug delivery systems in the literature.

Matrix	Drug	Drug Delivery System	Relative Antibacterial Response*	Sustained Release Time	Ref.
CMC/MCM-41	Methylene blue	Hydrogel	2.75	72 h	[32]
CMC/MCM-41/ZnO	Tetracycline	Hydrogel	4.14	72 h	[45]
Bovine Serium Albumine	Amoxillin	Electrospun nanofibers	3	150 h	[53]
PAN/Agar	Ampicillin	Electrospun nanofibers	3.33	180 h	[54]
PCL	Amoxillin	Electrospun nanofibers	5.5	200 h	[55]
PCL	Cefazolin	3D printing Nanofibers	5.4	120 h	[56]
PLA/PBAT	Cloxacillin	Electrospun nanofibers	3.75	50 h	[57]
CA	Benzocaine	Electrospun nanofibers	-	5 h	[58]
Cellulose/SBA-15CM-Ca2-SBA(30%)	Chloramphenicol	Composite membranes	4.23	270 h	This work

* Relative antibacterial response = ratio of the diameter of the inhibition zone to the diameter of sample (discs).

## Supplementary Materials

The following are available online at https://www.mdpi.com/2073-4360/11/5/808/s1, Figure S1. Nitrogen adsorption–desorption isotherms and pore-size distribution curve of (a) pristine SBA-15, (b) calcined CM-U-SBA(30%), (c) calcined CM-Ca1-SBA(30%), (d) calcined CM-Ca2-SBA(30%) and (e) calcined CM-Ca3-SBA(30%). Figure S2. XRD patterns of (a) CM, (b) CM-Ca2-SBA(10%), (c) CM-Ca2-SBA(20%), (d) CM-Ca2-SBA(30%) and (e) SBA-15. Table S1. Release parameters of the power law model for chloramphenicol in cellulose-based membranes.
