# Supplementary material for: Engineering Sustainable Antimicrobial Release in Silica-Cellulose Membrane with CaCO3-Aided Processing for Wound Dressing Application
Source: Polymers (Basel). 2019 May 6;11(5):808. doi: 10.3390/polym11050808 (PMC6571907; doi:10.3390/polym11050808)
Supplement: Supplementary file 1 [file polymers-11-00808-s001.pdf]

Supplementary Information

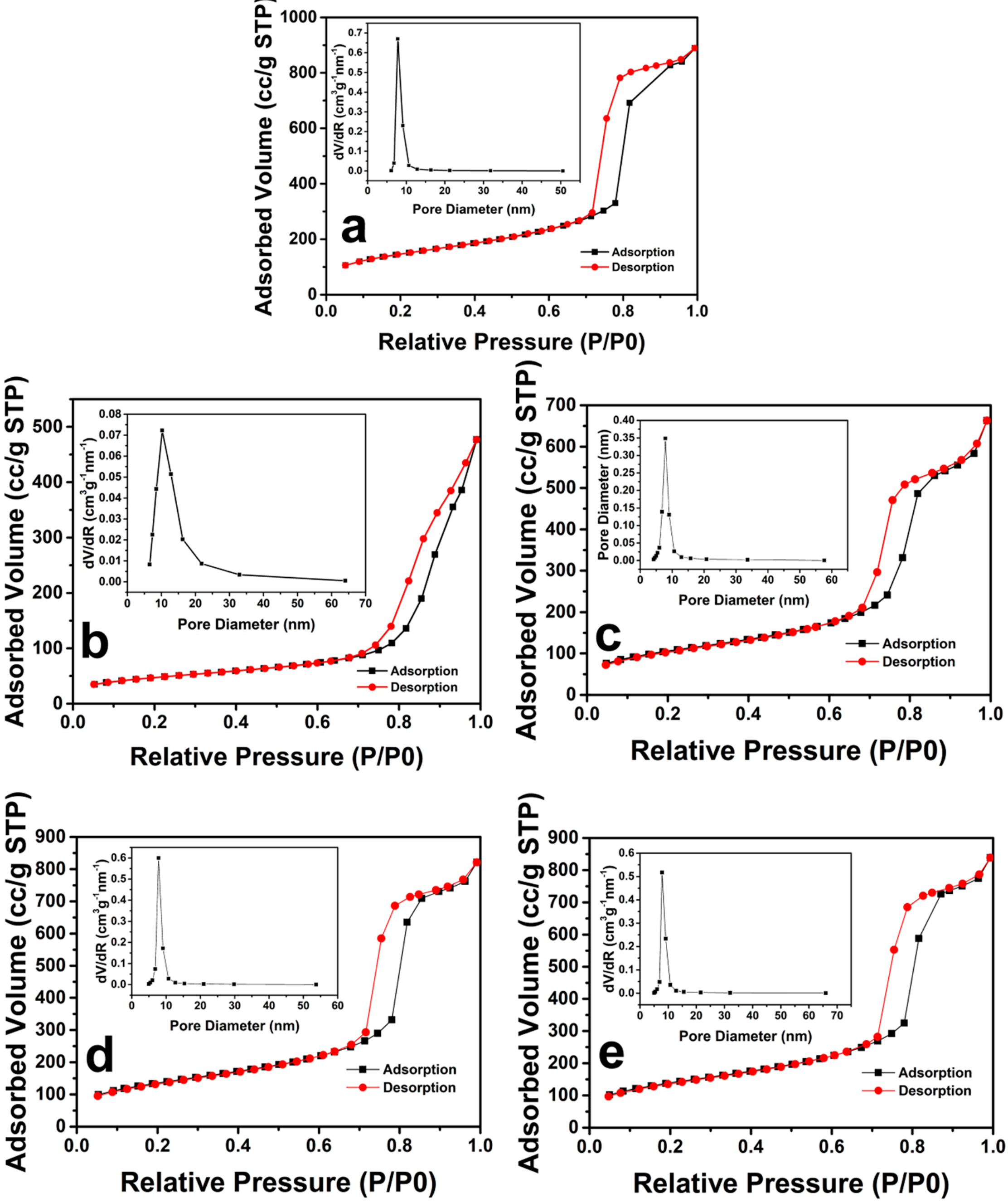

Figure S1. Nitrogen adsorption-desorption isotherms and pore size distribution curve of (a) pristine SBA-15, (b) calcined CM-U-SBA (30%), (c) calcined CM-Ca1-SBA(30%), (d) calcined CM-Ca2-SBA(30%) and (e) calcined CM-Ca3-SBA(30%).

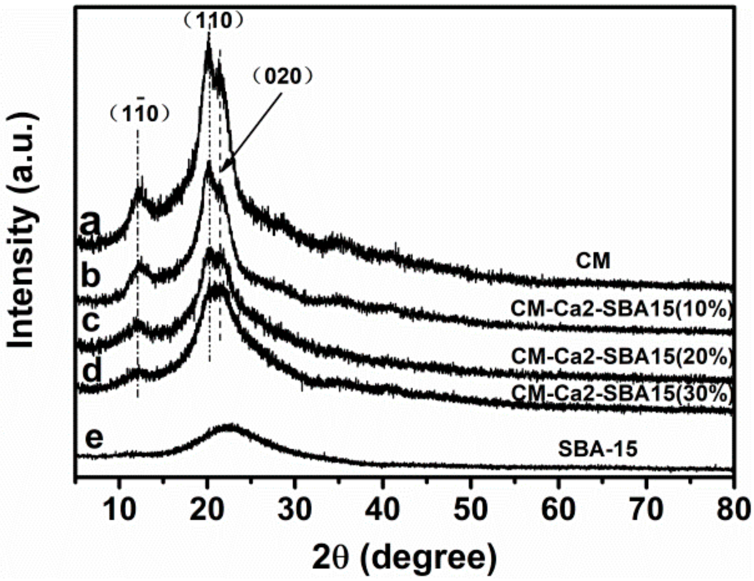

Figure S2. XRD patterns of (a) CM, (b) CM-Ca2-SBA(10%), (c) CM-Ca2-SBA(20%), (d) CM-Ca2-SBA(30%) and (e) SBA-15.

Table S1. Release parameters of power law model for chloramphenicol in cellulose based membranes

Sample	*n*	*K*	R^2
CM	0.240	0.459	0.988
CM-U-SBA(30%)	0.258	0.271	0.974
CM-Ca1-SBA(30%)	0.389	0.117	0.999
CM-Ca2-SBA(30%)	0.438	0.074	0.979
CM-Ca3-SBA(30%)	0.443	0.069	0.977
CM-Ca2-SBA(20%)	0.434	0.075	0.977
CM-Ca2-SBA(10%)	0.417	0.097	0.985
